# Effect of high-flow nasal therapy during exercise training in COPD patients with chronic respiratory failure: study protocol for a randomised controlled trial

**DOI:** 10.1186/s13063-019-3440-2

**Published:** 2019-06-08

**Authors:** Michele Vitacca, Irene Pietta, Marta Lazzeri, Mara Paneroni, on behalf of Associazione Italiana Riabilitatori Insufficienza Respiratoria (ARIR) and Associazione Italiana Pneumologi Ospedalieri (AIPO) rehabilitation group

**Affiliations:** 1 Istituti Clinici Scientifici Maugeri IRCCS, Respiratory Rehabilitation of the Institute of Lumezzane, Brescia, Italy; 2 ARIR Associazione, Milan, Italy; 3 Ospedale Niguarda Ca’Granda, Milan, Italy

**Keywords:** Rehabilitation, Exercise tolerance, Oxygen, Outcome

## Abstract

**Background:**

The benefit of pulmonary rehabilitation (PR) in symptomatic chronic obstructive pulmonary disease (COPD) is well known. However, advanced patients with chronic respiratory failure (CRF), a category excluded from most studies, are frequently unable to sustain a work-load sufficiently high to obtain the full benefit of PR on exercise tolerance. Recent studies involving heated and humidified high flow oxygen therapy (HFOT) showed positive effects on breathing pattern and ventilatory efficiency during effort. We thus plan to compare, in COPD patients with CRF undergoing a high-intensity exercise programme, the effect of using HFOT versus standard oxygen delivery via Venturi Mask (V-mask), at the same inspiratory oxygen fraction, on improving exercise endurance.

**Methods/Design:**

This is a multicentre randomised controlled trial that will involve 156 COPD inpatients with CRF recruited from seven PR hospitals. Patients will be randomised to one of two groups – V-mask versus HFOT. All patients will undergo the same high-intensity exercise programme using either of the oxygen delivery devices as per their group allocation. Training will consist of 20 sessions, over 1 month (5 sessions per week) within the hospitalisation period. Anthropometric and clinical data, including body mass index, diagnosis, spirometry and comorbidities (Cumulative Rating Scale) will be collected at baseline. At baseline and at the end of the exercise programme (primary assessment time) evaluation will include exercise tolerance (Constant Work Rate Exercise Test) (primary outcome), functional capacity (6-min walk test), maximal inspiratory pressure/maximal expiratory pressure, peripheral muscle strength (biceps and quadriceps) by manual dynamometer, respiratory exchanges (blood gases analysis), disability (Barthel Index and Barthel Dyspnoea Index), impact of disease (COPD Assessment test), and quality of life (Maugeri Respiratory Failure Scale-26). At the end of the training period, patient satisfaction will be evaluated.

**Discussion:**

This study will add knowledge about the exercise response in advanced COPD with CRF and verify if an alternative tool, namely HFOT, can increase the benefit obtained from PR.

**Trial registration:**

ClinicalTrials.gov ID NET03322787

Registered: 6 November 2017

**Electronic supplementary material:**

The online version of this article (10.1186/s13063-019-3440-2) contains supplementary material, which is available to authorized users.

## Background

Chronic obstructive pulmonary disease (COPD) is a heterogeneous disorder characterised by dysfunction of the small and large airways as well as by destruction of the lung parenchyma in highly variable combinations [1]. Dyspnoea and exercise intolerance are the most common symptoms in COPD and progress relentlessly as the disease advances. Increased dyspnoea leads to inactivity and consequent peripheral muscle deconditioning, resulting in a vicious cycle leading to further inactivity, social isolation, anxiety, depression and reduced quality of life [2–5]. Pulmonary rehabilitation (PR), with aerobic exercise training as a core component, is proposed in severe and very severe COPD, and has been shown to improve exercise capacity and quality of life [6]. The effectiveness of PR on exercise tolerance, dyspnoea and quality of life has already been demonstrated in patients with chronic respiratory failure (CRF) [7]. Nevertheless, these patients are frequently unable to sustain a workload high enough to obtain full benefit from the training programme [8]. CRF patients, in fact, are characterised by an unbearable exercise-induced dyspnoea, mainly due to an imbalance between ventilatory capacity and ventilatory demand [9], wherein ventilation during effort shows an out-of-proportion increase and so they prematurely reach the ventilatory reserve and are thus forced to stop exercising. The increase of dead space is the mechanism underlying this phenomenon [10]. Optimisation of drug therapy [11] along with non-pharmacological strategies can be useful to enhance exercise tolerance by reducing dyspnoea and work of breathing in advanced COPD with CRF. Among these techniques, oxygen supplementation delivered by nasal probes [12] and several different types of non-invasive ventilation (NIV) have been proposed [5]. In particular, NIV has the potential to reduce exercise dyspnoea by unloading respiratory muscles, allowing for higher levels of exercise intensity [5], but it is not always tolerated during exercise due to a variety of side effects such as claustrophobia, skin rash or eye irritation; furthermore, its use requires high expertise and is time-consuming for health professionals during exercise sessions [13]. High-flow nasal therapy (HFNT) is an emerging technique that may also be used to enhance ventilation and simultaneously provide an extended range of oxygen concentrations. It can deliver up to 60 L/min of heated, humidified air via nasal cannula, with or without additional oxygen. Above a flow of 20 L/min, HFNT can generate a positive pressure in the upper airways proportional to the set flow [14]. At rest, HFNT has been demonstrated to increase alveolar ventilation and reduce respiratory rate and tissue carbon dioxide while increasing tidal end-expiratory lung volumes and gas exchange, and reducing work of breathing in patients with COPD [15, 16].

To our knowledge, only one physiological cross-over study has shown that HFNT can increase exercise tolerance in patients with severe but stable COPD [17], but no clinical trials have investigated the effects of HFNT in a programme of exercise training for COPD with CRF. This treatment could represent an innovative alternative mode of NIV able to enhance exercise tolerance in these patients and help them during exercise training by decreasing dyspnoea. At the same time, this new technique could be proposed to increase training to a wider range of patients.

## Aims

### Primary aim

The primary aim of the trial will be to test the effect of using HFNT compared to a standard oxygen therapy delivered by the Venturi-mask (V-mask) at the same inspiratory fraction of oxygen (FiO_2_) in severe COPD patients with CRF performing 20 sessions (over 1 month) of exercise training. The efficacy of using HFNT will be evaluated in terms of exercise tolerance improvement.

### Secondary aims

The secondary aims will be to describe baseline factors associated to exercise tolerance improvement following exercise training or early training interruption.

## Methods

This is a multicentre randomised controlled study. Patients will be recruited from seven Italian respiratory rehabilitation hospitals, namely Istituti Clinici Scientifici Maugeri (Lumezzane (Brescia), Pavia, Tradate (Varese), Cassano Murge (Bari, Apulia), Veruno (Novara)), Villa Pineta Casa di Cura (Pavullo Nel Frignano, Modena) and Don Gnocchi Foundation IRCCS (Milano).

**Fig. 1:**
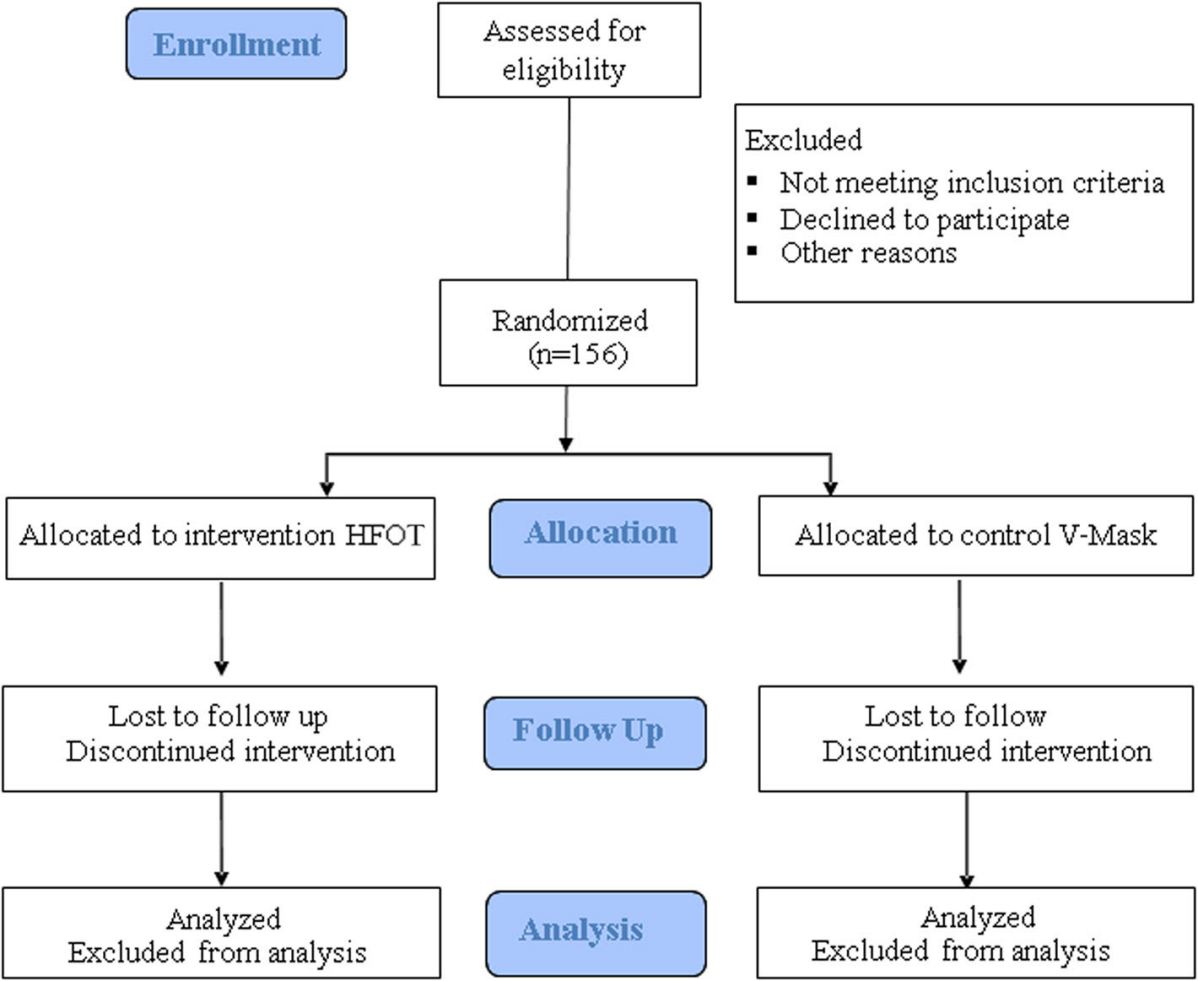
Study flow chart

A flow chart of the study design is shown in Fig. 1. The Standard Protocol Items of Recommendations for Interventional Trials (SPIRIT) Checklist is included in Additional file 1. The protocol was approved by the Ethics Committees of each centre and registered in ClinicalTrials.gov ID NET03322787. Participants will be provided, by the physiotherapist and pulmonologist involved in the study, oral and written information regarding the organisation of the study and written informed consent will be obtained from each participant.

### Study population

Patients will be eligible for inclusion if they fulfil the following criteria: (1) diagnosis of COPD (post bronchodilator spirometry values: forced expiratory volume in 1 s (FEV1)/forced vital capacity (FVC) < 0.7) [18]; (2) long-term oxygen therapy prescription for at least 3 months; (3) clinical stability (pH range 7.38–7.42, no change in respiratory drug therapy within the previous 7 days); and (4) provision of informed consent. Patients will be excluded if they have orthopaedic or neurological disease that could limit exercise performance, cognitive impairment (Mini-Mental State Examination < 22) [19], heart diseases, pulmonary fibrosis or obstructive sleep apnoea syndrome. Table 1 summarises the eligibility criteria. A trained physiotherapist will screen the patient lists every day at each rehabilitation hospital with the aim to detect eligible candidates for the study and will check their eligibility in terms of the inclusion and exclusion criteria. If patients seem appropriate, the physician of each department will inform them about protocol; patients will have 24 h to consider their decision. Patients will be then enrolled and randomised to either the intervention (HFNT) or control (V-mask) group.

**Table 1:** Inclusion and exclusion criteria

Inclusion criteria	Exclusion criteria
1. Chronic obstructive pulmonary disease + chronic respiratory failure 2. Long-term oxygen therapy 3. Clinical stability (7.35 < pH < 7.46)	1. Orthopaedic or neurological disease 2. Cognitive impairment (MMSE < 22) 3. Anamnestic history of ischemic heart disease or chronic heart failure 4. Pulmonary fibrosis

*MMSE* Mini-Mental State Examination

### Randomisation

The participants will be randomised in variable blocks by centres. Enrolled patients will be randomised to either an intervention or a control group (randomisation ratio 1:1). The allocator will be a researcher not involved in the study, blinded on group allocation. The allocator will generate a randomisation list by a dedicated software (https://www.randomizer.org/). When a new patient (after control of eligibility and signing of informed consent) will need to be enrolled, care providers from each centre will contact the randomisation centre by phone for definitive allocation. Patients will be aware of their allocation.

### Intervention protocol

**Fig. 2:**
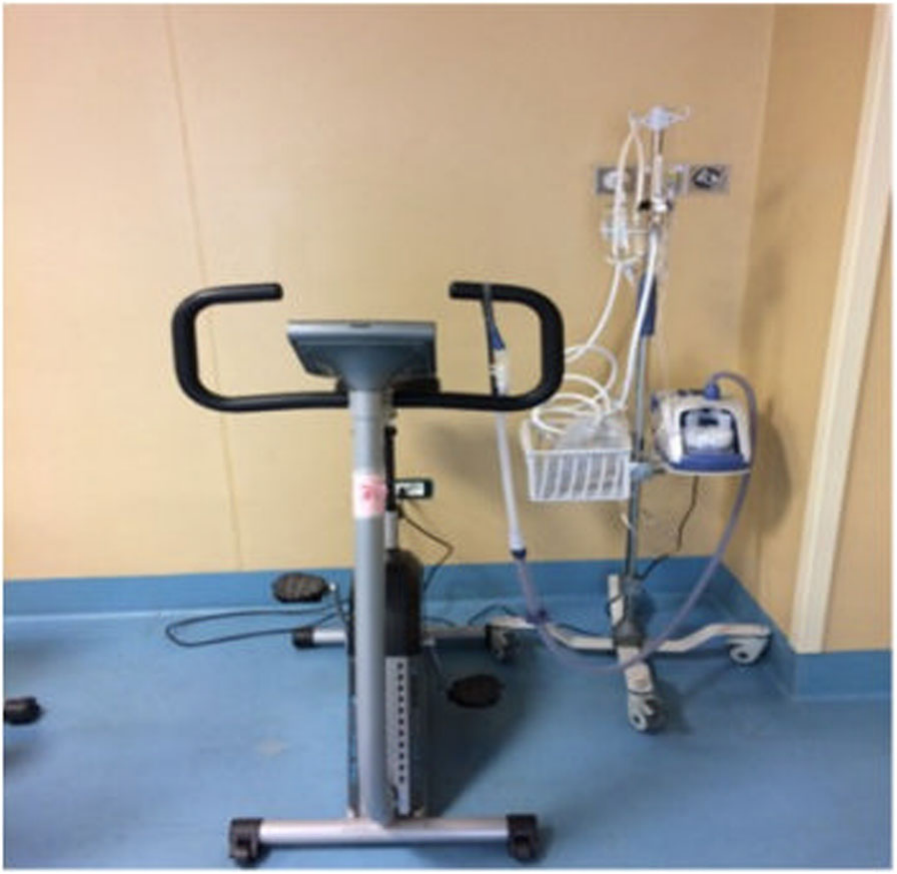
Description of the training setting of the intervention group

Two different researchers will be involved in the study; the first will follow the patient’s training and the second (assessor) will evaluate the outcome measures. Only the assessor will be blind to patient allocation (single blind trial). Figure 2 shows the training setting of the intervention group.

#### Run-in phase

In order to define the necessary O_2_ to train patients before the start of the training programme, all patients will undergo a 30-min run-in training session on a cycle ergometer at an intensity corresponding to 50% of the predicted maximum exercise workload (Luxton equation) [20]. Patients will perform a preliminary session breathing through the V-mask (Fiab S.p.a. ISO 13485, Italy) and the respiratory therapist will note the FiO_2_ able to maintain blood oxygen saturation (SpO_2_) constantly up to 93%.

#### Training programme

All patients will perform a cycle ergometer endurance training programme consisting of at least 20 training sessions lasting 30 min each, within a 1-month time period (5 sessions per week). The sessions will start with a warm-up period of 0 W, then patients will train at the intensity prescribed. The intensity will start from 50% of the theoretical maximal load estimated by Luxton’s equation [20] based on distance walked in the 6-min Walking Test (6MWT). Increase or reduction of intensity of the training will be decided following the protocol of Maltais et al. [21]. In brief, we will increase the cyclo-ergometer workload by 10 W when patients refer dyspnoea and muscular fatigue evaluated by a Borg score of less than 4 [22] in the previous session. Workload will remain the same for scores between 4 and 5 [21] and will be decreased for scores beyond 5.

Patients will be asked to maintain a cycling rate between 50 and 60 rpm and to breathe as quietly as possible. A trained respiratory therapist will constantly supervise the rehabilitative intervention. During the protocol period, patients will use medications and oxygen therapy at rest as prescribed. During training, patients will be supplemented with oxygen therapy delivered in two different ways (HFNT or V-mask); the modality of oxygen delivery will define the allocation group.

### Allocation

#### Intervention group (oxygen delivered by HFNT)

The training will be done while using the HFNT device. HFNT will be administered using the AIRVO™ 2 device (Fisher & Paykel, Auckland, New Zealand). This system generates humidified, reheated air (between 31 °C and 37 °C) up to 60 L/min FiO_2_ by altering the O_2_ supplementation within the system (0.21–1 FiO_2_) [23]. The air will be administered with an open-circuit through Optiflow™ nasal cannula (Fisher & Paykel). The Optiflow™ nasal cannula will deliver a gas flow directly into the nares without gas jetting; it has soft and flexible nasal prongs, and an adjustable head strap which fits over the patient’s ear, allowing freedom of patient movement. The cannula will be connected to the machine by a delivery tube with an inner breathable film that reduces the condensates. Air-flow will be set at the highest value tolerated by the patient starting from the maximum value of 60 L/min permitted by the instrument and will be reduced in case of intolerance. In the same way, the temperature will be set at 37 °C and be reduced in case of intolerance. Every change in flow and temperature will be recorded. FiO_2_ will be set according to the run-in phase.

#### Control group (oxygen delivered by V-mask)

The training will be performed using the V-mask (Fiab S.p.a. ISO 13485, Italy) with the FiO_2_ set during the run-in session. The V-mask is a certificated medical device that delivers a known oxygen concentration to patients on controlled oxygen therapy. The mask includes color-coded diluters, each engineered to deliver a set amount of oxygen to the mask. Masks are not made with natural rubber latex.

### Outcome measures

At baseline (T0), anthropometric parameters, e.g. age, sex, body mass index, diagnosis, spirometry (FEV1, FVC, FEV1/FVC) [24] and comorbidities (using Cumulative Illness Rating Scale [25]), will be recorded. All other outcome measures described below will be assessed at T0 and at the end of the study period (T1, primary assessment time).

#### Primary outcome

Exercise tolerance by the Constant Work Rate Exercise Test (CWRET). The time to exhaustion of this test will be the primary outcome of the study. The CWERT will begin with a 2-min warm-up without resistance followed by a phase of exercise. The workload will be set at 80% of maximal load predicted according to the Luxton equation [20] using 6MWT performed at T0. Patients will be monitored by oximeter (Nonin2500A-Nonin Medical Inc., Plymouth, USA) and by one-lead electrocardiogram telemetry (DS5700, Fukuda Denshi Ltd. Co, Tokyo, Japan). Patients will be required to maintain a peddling frequency of 60–65 revs/min [26]. The test will end when the modified Borg dyspnoea or fatigue scale [22] reach level 8 or it will be stopped by the respiratory therapist if SpO_2_ decreases below 80% or heart rate surpasses the maximal heart rate predicted under effort. The test will also be interrupted if any of (1) ST segment depression, (2) angina pectoris, or (3) malignant arrhythmias (e.g. ventricular tachycardia, atrial fibrillation) occur. Arterial blood pressure, heart rate and SpO_2_, and the modified Borg Dyspnoea and Fatigue scale at the beginning and at the end of the test, will be recorded. Time to exhaustion will be recorded using a stopwatch. The exercise tolerance time will be the sum of the warm-up period and test phase. After the test, patients will be given a 3-min active recovery phase without resistance on the cycle ergometer. Patients will breathe with their usual oxygen flow through nasal prongs during both 6MWT and CWRET.

#### Secondary outcomes

1. Functional capacity by the 6MWT carried out according to American Thoracic Society/European Respiratory Society recommendations [27].
2. Respiratory muscle strength will be evaluated by maximal inspiratory pressure and maximal expiratory pressure. An electronic manometer (Precision Medical, Northampton, USA) will be used according to international recommendations [28].
3. Peripheral muscle strength (biceps and quadriceps) will be evaluated by manual dynamometer (Chatillon^®^ X-3328Series, Ametek Inc., Florida, USA). The measure will be conducted using a hand-held dynamometer according to the position tests described by Andrews et al. [29]. For biceps, we will position patients sit on a chair, with adducted arm and elbow at 90° of flexion. We will ask patients to perform an elbow flexion and the dynamometer will be fixed at wrist level. For quadriceps, we will position patients sit on a high chair and ask them to perform a knee extension placing the dynamometer just over the ankle.
4. Dyspnoea evaluation by the Medical Research Council scale [30].
5. Respiratory exchanges by blood gas analysis. We will record the partial pressures of O_2_, CO_2_, O_2_/FiO_2_, and pH.
6. Disability, measured by the Barthel Index [31] and Barthel Dyspnea Index [32].
7. Impact of disease measured by the COPD Assessment test [33].
8. Quality of life by the Maugeri Respiratory Failure Scale-26 [34], specifically dedicated to CRF patients.

**Fig. 3:**
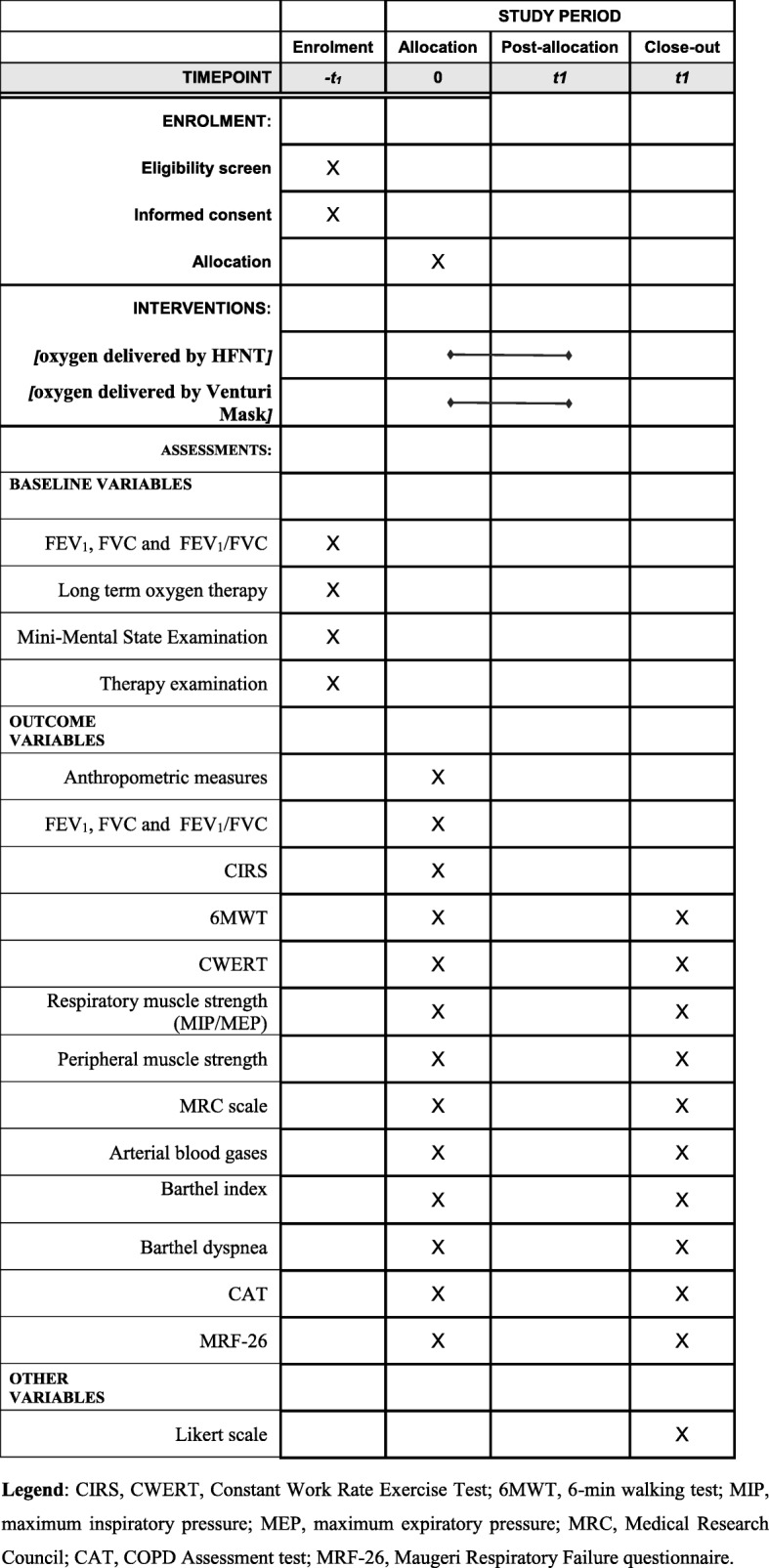
SPIRIT checklist, describing the outcome measures and the time-points of evaluation

Global patient satisfaction with the training sessions and oxygen device will be rated on a 5-point Likert scale [35] using a bespoke questionnaire. We will measure the satisfaction score the day after the training programme conclusion. We will ask patients the following questions: “How would you rate your feeling of comfort and well-being during training?” (0 = I think that it was not sufficient; 1 = sufficient; 2 = good; 3 = a bit heavy, 4 = too heavy for my physical condition); “What do you think about the oxygen delivery device used?” (0 = not sufficient (several problems), 1 = sufficient, 2 = good, 3 = very good, 4 = excellent). Adverse events that occur during exercise sessions and during hospital admission course will be registered. The outcome measures and the time-point of evaluations were described with the SPIRIT checklist (Fig. 3).

### Statistical analysis

#### Sample size

We propose a sample size based on an effect size of treatment of 130 s of difference in seconds of CWRET between the groups. The estimated improvement (mean and standard deviation) in the control group was calculated in relation to previous studies [36], defining 150 s as the minimally clinically important difference of improvement of CWRET after rehabilitation. The improvement in the treatment group was estimated by an internal pilot study conducted in 10 patients. A total of 156 participants would provide 90% power to detect 130 s (SD 250) difference between the groups, with 5% significance. Due to a high expected drop-out rate (patients transferred to acute care, severely ill with fever, severe dyspnoea, acute respiratory failure, etc.) we will enrol at least 170 patients. We expect a recruitment rate of at least 23 patients per centre.

#### Analysis plan

Details about project start-up phase and patient recruitment are reported in ClinicalTrials.gov ID NET03322787. Statistical analysis will be performed using STATA 11 (StataCorp LLC, Texas 77845–4512 USA).

Continuous variables with normal distribution will be presented as mean ± standard deviation (SD), if non-normally distributed, as median and interquartile range. The distribution of continuous variables will be verified by the Shapiro–Wilk test. Binary and categorical outcomes will be described using frequencies and percentages in each group.

The primary analysis will be a modified intention-to-treat analysis, including all randomly assigned participants for whom data on the primary endpoint is available. Secondary analysis will be conducted per-protocol (all completers).

Primary analysis of the primary outcome will be through a linear regression model considering the outcome measures at the end of the training as a dependent variable and intervention/control as an explanatory variable with adjustment for the baseline value of the primary outcome. The model will also take into account the centre as a stratification variable.

The baseline, follow-up values and the unadjusted change in outcome measures between baseline and the end of study for both groups will be also presented.

Secondary analysis will consist of a two-sample *t* test comparing differences in the changes in all outcome measures after rehabilitation between the intervention and control groups.

In order to define which baseline characteristics are related to the improvement of primary outcome and to drop-out event, we will perform an explanatory data evaluation analysing the odds ratio (with corresponding 95% confidence intervals and *P* values) of improvement/non-improvement on CWRET above 150 s [36] and the completion/dropout of the programme for each baseline variable. A *P* value of < 0.05 will be considered statistically significant.

### Data sharing

Individual participant data that will underlie the results to be reported in the results article, after deidentification (text, tables, figures and appendices) will be available for meta-analysis after article publication. Those interested will be able to request the data from the corresponding author by mail.

### Dissemination of findings

Authorship on peer-reviewed manuscripts will be offered to those who have made a significant contribution to the conception or design of the project or the acquisition, analysis, or interpretation of data for the work, and/or drafted the work or reviewed it critically for important intellectual content. There are no publication restrictions for this research. Dissemination of results will be via traditional channels of journal publications and scientific conferences.

## Discussion

Aerobic exercise training in severe and very severe COPD with chronic respiratory failure has been not well studied [6, 7] despite high workload, unbearable exercise-induced dyspnoea [9] and frequent premature interruption of exercise creating a significant limitation to exercise for these patients. Non-pharmacological strategies such as NIV have been proposed [5] to reduce this discomfort, with inconclusive and variable results due to poor tolerance and the high expertise and time-consumption required from health professionals during exercise sessions [13]. Theoretically, HFNT could obtain similar results (in terms of exercise tolerance) with less discomfort and time required.

The use of the HFNT device will not change the underlying disease process, but we expected that (at least for a subgroup of patients) its use during moderate or high intensity training will further improve functional capacity, exercise tolerance, respiratory and peripheral muscle strength, blood gases, disability, symptoms, impact of disease and quality of life, with high patient satisfaction. Future studies may investigate the applicability and benefits of the routine application of this device in severe COPD and CRF.

## Additional file

Additional file 1: SPIRIT 2013 Checklist. (DOC 123 kb)

## Data Availability

During the time course of the study, the database will be closely related to the principal investigator and managed by the data manager. All data generated or analysed during this study will be included in the subsequently published results article (and its supplementary information files).

## Abbreviations

6MWT: 6-min Walking Test
COPD: chronic obstructive pulmonary disease
CRF: chronic respiratory failure
CWRET: Constant Work Rate Exercise Test
FEV1: forced expiratory volume in 1 s
FiO_2_: inspiratory fraction of oxygen
FVC: forced vital capacity
HFNT: high-flow nasal therapy
NIV: non-invasive ventilation
SpO_2_: blood oxygen saturation
T0: baseline
V-mask: Venturi-mask
WOB: work of breathing
